# Malignancy‐associated ischemic stroke: Implications for diagnostic and therapeutic workup

**DOI:** 10.1111/cns.14619

**Published:** 2024-03-26

**Authors:** Wanqing Xie, Szuyao Hsu, Yuxuan Lin, Lv Xie, Xia Jin, Ziyu Zhu, Yunlu Guo, Caiyang Chen, Dan Huang, Johannes Boltze, Peiying Li

**Affiliations:** ^1^ Department of Anesthesiology, Renji Hospital Shanghai Jiao Tong University School of Medicine Shanghai China; ^2^ School of Life Sciences University of Warwick Coventry UK; ^3^ Clinical Research Center, Renji Hospital Shanghai Jiao Tong University School of Medicine Shanghai China; ^4^ Outcomes Research Consortium Cleveland Ohio USA

**Keywords:** anticoagulation, biomarkers, cancer, ischemic stroke, malignancy, reperfusion therapy

## Abstract

**Background:**

Patients with malignancies have an increased risk of suffering ischemic stroke via several mechanisms such as coagulation dysfunction and other malignancy‐related effects as well as iatrogenic causes. Moreover, stroke can be the first sign of an occult malignancy, termed as malignancy‐associated ischemic stroke (MAS). Therefore, timely diagnostic assessment and targeted management of this complex clinical situation are critical.

**Findings:**

Patients with both stroke and malignancy have atypical ages, risk factors, and often exhibit malignancy‐related symptoms and multiple lesions on neuroimaging. New biomarkers such as eicosapentaenoic acid and blood mRNA profiles may help in distinguishing MAS from other strokes. In terms of treatment, malignancy should not be considered a contraindication, given comparable rates of recanalization and complications between stroke patients with or without malignancies.

**Conclusion:**

In this review, we summarize the latest developments in diagnosing and managing MAS, especially stroke with occult malignancies, and provide new recommendations from recently emerged clinical evidence for diagnostic and therapeutic workup strategies.

## Graphical abstract

Eary identification and recomended interventions of stroke with occult malignancies.

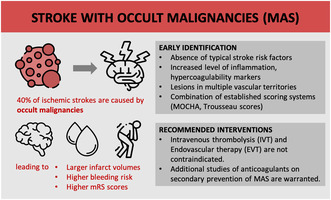

## 1 INTRODUCTION

Stroke is the major cause of disability and death worldwide.^1^, ^2^, ^3^ The global stroke burden has increased from 1999 to 2013 despite slightly declining incidence and mortality.^4^ Ischemic stroke can be classified into five major categories according to the Trial of ORG 10172 in Acute Stroke Treatment (TOAST) criteria: (1) large‐artery atherosclerosis, (2) cardioembolism, (3) small‐vessel occlusion, (4) stroke of other determined etiologies and (5) stroke of undetermined etiology. Active malignancy is discussed as a potential cause of stroke of undetermined etiology. According to a nationwide study in the United States, about 1 of 10 hospitalized ischemic stroke patients has comorbid malignancy.^5^ In turn, approximately 15% of patients with malignancies suffer from cerebrovascular diseases.^6^ About 40% of ischemic strokes in these patients are of cryptogenic etiology.^7^, ^8^ Patients with malignancies have a 2.2 times higher risk of suffering from ischemic stroke than patients without malignancies according to a Swedish nationwide follow‐up study.^9^ Stroke can occur at any stage of malignancy, and both occult (relative risk, 1.75/2.00) and manifest malignancies (relative risk, 1.30/1.41) increase the risk of ischemic stroke.^10^ Furthermore, risk of arterial thromboembolic events such as myocardial infarction and ischemic stroke peak about 1 month prior to the diagnosis of malignancy, further highlighting the close relationship between stroke and occult malignancies.^11^ Hence, malignancy‐associated ischemic stroke (MAS) is attracting increasing attention as a subtype of ischemic stroke. MAS not only includes stroke caused by cancer‐related hypercoagulable state but also incidental stroke caused by common causes, stroke related to tumor emboli, stroke related to tumor direct invasion of blood vessels, and stroke related to cancer treatment.

Etiologically, MAS can be divided into three types: (i) direct tumor effects such as tumor emboli, (ii) coagulopathy such as nonbacterial thrombotic endocarditis, and (iii) iatrogenic effects including chemotherapy, radiation therapy, and surgery‐induced vascular injuries.^12^, ^13^, ^14^ Moreover, MAS patients are more likely to have larger infarct volumes, higher bleeding risk, worse short‐term prognosis such as more frequent deep vein thrombosis and pulmonary embolism, as well as deteriorated long‐term prognosis with higher 90 day‐recurrence rate and death rate.^10^, ^15^, ^16^, ^17^, ^18^ Patients suffering from severe MAS often require palliative care.^19^ Given the emerging clinical significance of MAS, there is a lack of a comprehensive summary or practical clinical guidance for the diagnostic treatment workup of MAS.

In this review, we focus on recent advances in the identification of high‐risk MAS patients, characteristic features of MAS, followed by comparison of treatment and prevention options of MAS patients. The short‐term as well as long‐term prognosis of MAS patients is also discussed.

## 2 DIAGNOSTIC CLUES OF MAS PATIENTS

### 2.1 Baseline characteristics of patients with MAS

The clinical features of cryptogenic stroke in MAS patients are often different from those in patients without malignancies. MAS patients tend to be in worse conditions^20^ and are more likely to exhibit altered mental status, aphasia, and limb weakness ipsilateral to the stroke along with malignancy‐associated symptoms, such as weight loss, fever, hematochezia, melena, and adenopathy.^21^, ^22^ In addition, MAS patients tend to have fewer atherosclerotic risk factors (compared to other stroke patients), higher plasma D‐dimer levels, and more multiple vascular lesions than stroke patients without malignancy.^23^ Coagulopathies and a cachectic state caused by malignancies could affect the ischemic stroke outcome in MAS patients.^19^, ^23^ Therefore, stroke patients exhibiting at least one of the mentioned features should undergo a thorough examination to exclude the presence of occult malignancies once acute stroke management is completed.

Nevertheless, demographic data differ from currently available studies. For instance, a retrospective study comparing 226 patients found that MAS patients tended to have fewer traditional risk factors of stroke like hypertension, hyperlipidemia, and atherosclerosis.^18^ However, other stroke‐related factors such as higher age, smoking history, and diabetes and/or venous thromboembolism were more frequently found in MAS patients as suggested by some prospective studies.^24^, ^25^ The discrepancies between the above two studies could be attributed to the less specified definition of cryptogenic stroke, including different patient populations as compared to that of conventional stroke. In another study that included 348 cryptogenic stroke patients with (*n* = 71) and without active malignancies (*n* = 277), vascular risk factors, hypertension, and hyperlipidemia were less prevalent in those with active malignancy, while other factors, including demographic profiles, such as history of diabetes, smoking, and coronary artery diseases, and pre‐stroke medications, did not differ between the two groups.^26^ Data from the Danish Stroke Registry indicate that stroke patients with occult malignancy are often relatively young (40–50 years of age),^24^ more likely to be female,^7^ and have a higher prevalence of deep vein thrombosis or microembolic events than those without malignancy, which may also partially explain the absence of conventional stroke risk factors in MAS.

Emerging evidence suggests that atrial fibrillation (AF), especially recently onset AF, often precedes the diagnosis of malignancies.^27^, ^28^, ^29^, ^30^ A population‐based study including over 24,000 MAS patients reported an incidence of newly diagnosed AF of 1.8%.^31^ Malignancies of the colon were most strongly associated with AF among the malignant subtypes examined.^32^ Although the underlying mechanisms of the increased long‐term risk of AF in individuals with malignancy remain largely unknown, there are some preliminary studies suggesting that higher age, systemic inflammation, and metabolic and endocrine abnormalities caused by a paraneoplastic environment could contribute to the occurrence of AF in these patients.^33^, ^34^

### 2.2 Neuroimaging of MAS patients often detect multiple cerebral lesions

Neuroimaging, such as computerized tomography (CT) and magnetic resonance imaging (MRI), are crucial for the diagnosis of ischemic stroke.^35^, ^36^, ^37^ MRI can differentiate cerebrovascular pathology from infection, trauma, or cerebral malignancies, as well as discover “silent” strokes.^38^, ^39^ Multiple lesions in different vascular territories have been found in diffusion‐weighted imaging (DWI) in patients with cryptogenic stroke and known malignancies.^26^, ^35^ Importantly, the number of blood supply territories involved can independently indicate occult malignancy. More than one territory involved could indicate occult malignancy, with a maximum Youden's index (Appendix S1) of 0.56,^40^ meaning that patients with more than one territory involved on DWI‐MRI may need extensive screening for occult systemic malignancy. Additionally, the so‐called “three territory sign” (DWI lesions involving bilateral anterior and posterior circulation, being nonenhancing, nonring‐appearing clusters or single areas of restricted diffusion of 0.5–2 cm with a peripheral location or larger vascular territories, uncommonly in a watershed distribution, and with absence of diffuse cortical ribbon or deep gray nuclei involvement) was proposed to provide a specific diagnostic clue of malignancy‐associated hypercoagulation, showing a low sensitivity of 23.4% but a high specificity of 96.4%.^41^, ^42^

### 2.3 Current biomarkers and the future development of new diagnostic tools of MAS

Identifying reliable MAS biomarkers could facilitate the screening of MAS patients. Higher levels of inflammatory markers, such as erythrocyte sedimentation rate (ESR) and C‐reactive protein (CRP); hypercoagulability markers, such as fibrinogen and D‐dimer; and tumor biomarkers, such as CA125/199 and lower levels of hemoglobin, are often seen in MAS.^18^, ^43^, ^44^ Increased fibrinogen (≥600 mg/dL) or CRP (≥20 mg/L) suggest increased risk of MAS (specificity of 96% and 91%, respectively).^45^ D‐dimers are particularly valuable as biomarkers for occult malignancy in stroke patients.^46^ D‐dimer levels are usually elevated in MAS patients with both malignancy and stroke, compared to those with malignancy or stroke only.^47^, ^48^ D‐dimer levels above 5.5 mg/L reliably predict MAS independently from MRI findings.^49^ Additionally, increased D‐dimer levels are associated with higher mortality in MAS patients.^43^, ^50^, ^51^, ^52^

New MAS biomarkers have been recently proposed based on mechanisms underlying malignancy‐related stroke, such as eicosapentaenoic acid (EPA), cancer cell‐derived extracellular vesicles, neutrophil extracellular trap (NET)‐specific biomarkers, decondensed chromatins, and blood mRNA. EPA is an omega‐3 polyunsaturated fatty acid involved in cellular homeostasis. It inhibits cancer initiation and progression.^53^ Significantly lower EPA levels were detected in patients with active malignancy and cryptogenic stroke (1.26 ± 0.72 vs. 1.89 ± 1.27 μmol/L; *p* = 0.02), independently of age and D‐dimer levels.^54^ The Optimal Anticoagulant Strategy in Stroke related to cancer (OASIS‐Cancer) study (ClinicalTrials.gov identifier NCT02743052) is ongoing to discover potential molecules such as cancer cell‐derived extracellular vesicles, procoagulant proteins, and microRNAs associated with MAS. Among NET‐specific biomarkers, plasma DNA (cell‐free DNA) and nucleosomes (assessed by ELISA using CLB‐ANA/58 and CLB‐ANA/60 antibodies) from decondensed chromatins are elevated in MAS patients,^55^ suggesting the correlation between NETosis and MAS. In a malignancy‐featured pathobiological environment, neutrophils are prone to NET formation and subsequent NETosis.^56^ Decondensed chromatins are prothrombotic and procoagulant, increasing the risk of deep vein thrombosis^57^ and MAS. However, it is uncertain whether the macromolecular structure of NETs and chromatin directly activates coagulation.^58^ Tumor‐derived extracellular vesicles may induce NET formation^59^ and thus facilitate MAS. Moreover, blood mRNA screening may aid early identification of MAS.^60^, ^61^, ^62^, ^63^ Blood mRNA profile analysis in MAS patients showed that interleukin‐1(IL‐1), interferon, relaxin, mammalian target of rapamycin (MTOR) signaling, sequestosome‐1(SQSTMI1), and cAMP response element binding protein‐1 (CREB1) were differentially expressed compared to stroke patients without malignancy.^64^ The above evidence suggests that different molecular pathways in autophagy, immunity, or inflammation are activated. Therefore, future studies should evaluate whether blood mRNA/DNA can aid the diagnosis and predict the prognosis of MAS.

Nevertheless, no single biomarker has shown sufficient diagnostic value for MAS due to the common hypercoagulability caused by stroke or the tumor itself and the complexity of hypercoagulability caused by malignancy treatment.^65^ Pro‐coagulant molecules can be produced by either tumor cells or immune cells during vascular injuries and tissue invasions,^12^, ^15^ resulting in a hypercoagulable environment in the blood stream. A predictive score for occult malignancy using the area under the receiver operating characteristics (AUC–ROC) curves and Bayes' theorem (including D‐dimer ≥3 mg/L, hemoglobin ≤12 g/dL, and smoking history) may identify MAS patients with up to 53% probability.^66^ The so‐called Trousseau score (after the Trousseau syndrome, an acquired blood clotting disorder associated with occult malignancy) can be used to differ MAS from other cryptogenic strokes (Table S1).^7^, ^67^ Five factors are included in the Trousseau score: high D‐dimer (≥10 mg/L), lesions in multiple territories, active cancer, low platelet counts (<150,000/μL), and female sex. A Trousseau score of over 3 suggested MAS and poor overall survival rate.^7^ The sensitivity of MOCHA (Markers of Coagulation and Hemostatic Activation) profiles, including D‐dimer, prothrombin fragment 1.2, thrombin–antithrombin complex, and fibrin monomer, can be used for aiding the etiological diagnosis in stroke patients. It is suggested that a normal MOCHA profile and left atrial size have a 100% sensitivity for ruling out atrial fibrillation, malignancies, venous thromboembolism, or other defined hypercoagulable states as causes for cryptogenic stroke.^68^ In addition to the clotting parameters that can be detected in the peripheral blood, clots retrieved by endovascular thrombectomy have also been explored as potential clues to determine stroke etiologies.^69^, ^70^ Retrieved clots from thrombectomy in MAS patients were rich in fibrin and platelets and had higher platelet and lower erythrocyte contents than those from patients without malignancies. This can help differentiate MAS from other etiologies such as cardioembolism or atherosclerosis.^71^, ^72^

**FIGURE 1:**
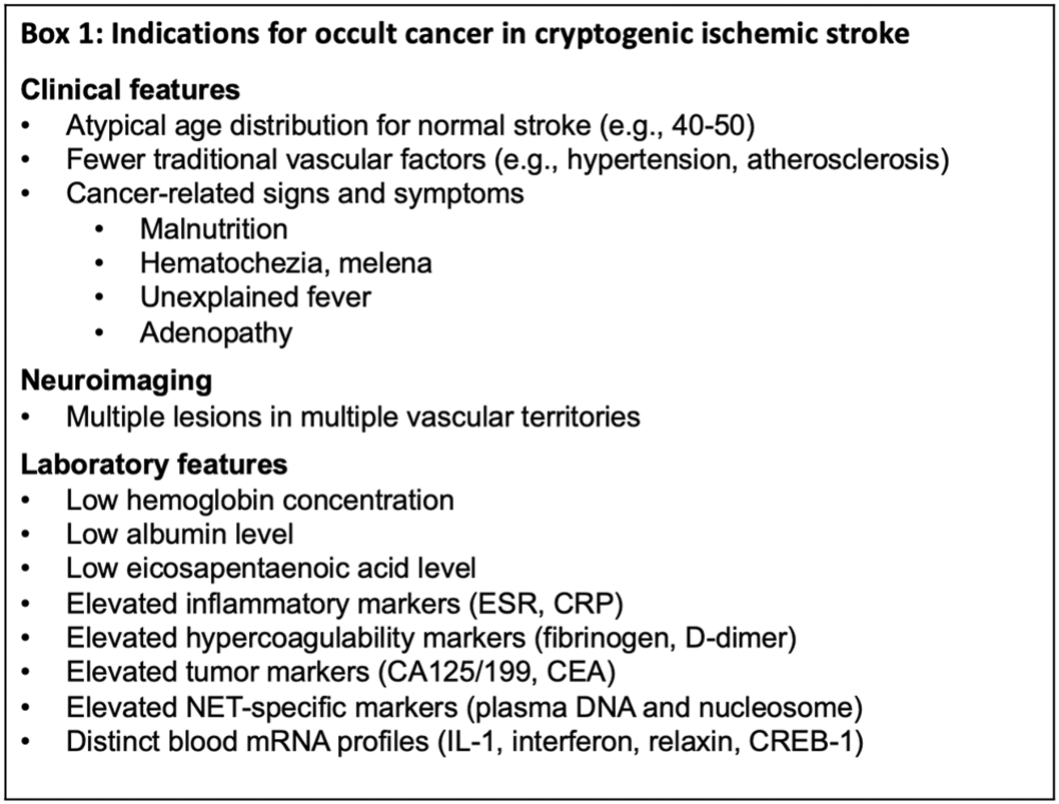
Indications for occult malignancy in acute ischemic stroke. Clinical symptoms, neuroimmaging, and laboratory features that are distinct in patients with malignancies, such as computerized tomography (CT), magnetic resonance imaging (MRI), and laboratory features such as hypercoagulation, conventional malignancy markers, and novel plasma biomarkers. CEA, carcinoembryonic antigen; CRP, C‐reactive protein; ESR, eythrocyte sedimentation rate; IL‐1, interlukin‐1.

**FIGURE 2:**
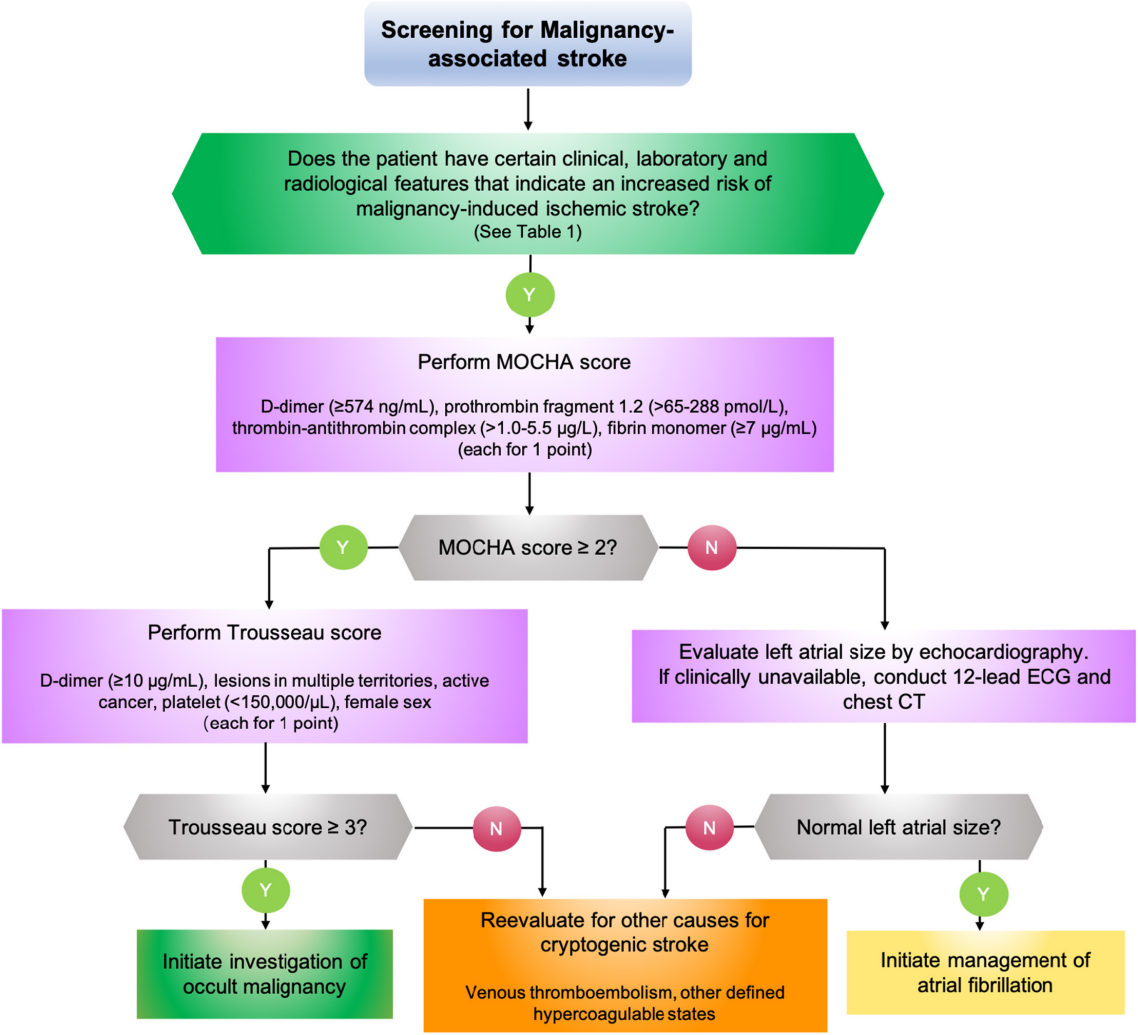
Clinical algorithm for malignancy‐associated stroke screening. Recommendations for discrimination of stroke etiologies and subsequent clinical management include initiating investigation of occult malignancy, reevaluating for other causes for cryptogenic stroke, and initiating management of atrial fibrillation. ECG, electrocardiography; MOCHA, markers of coagulation and hemostatic activation; N, no; Y, yes.

Overall, MAS patients present distinct features, as summarized in Figure 1. They are not likely to have typical age and risk factor profiles of ‘classical’ stroke patients but exhibit malignancy‐related features. Lesions in multiple vascular territories, especially the three‐territory sign, can indicate occult malignancy. Next to established biomarkers such as hypercoagulation and common malignancy markers, new plasma biomarkers such as EPA or mRNA/DNA profiles indicating autophagy, immunity, or inflammation hold the promise to predict occult malignancies. Furthermore, analysis of retrieved clots after stroke management can help discriminate stroke etiologies and guide subsequent clinical management. Combination of biomarkers can increase the sensitivity and specificity for the clinical application of scoring systems to help clinicians differentiate MAS from other causes for cryptogenic stroke (Figure 2).

Physicians should investigate potential occult malignancies once featured MAS characteristics in clinical, laboratory, and radiological signs are found. Contrast‐enhanced CT scanning of the chest, abdomen, and abdomen/pelvis should be done since lung, pancreatic, and colorectal cancer have been suggested as the most common malignancies in stroke patients.^1^, ^58^, ^73^ Moreover, age‐appropriate malignancy screening should also be conducted.^22^

## 3 THERAPEUTIC OPTIONS FOR MAS PATIENTS

Intravenous thrombolysis (IVT) is the standard treatment for acute ischemic stroke within a time window of 4.5 hours (nowadays often longer in patients with a penumbra) from symptom onset. IVT on average results in 25% reduction in disability.^69^, ^74^, ^75^ Specifically, patients with an INR < 1.7 while on warfarin or those with minor strokes benefit from IVT.^76^ For patients who are comorbid or exhibit large artery occlusions and a penumbra, endovascular therapy (EVT) can be applied within up to 24 h of onset.^77^ However, whether MAS patients can receive these treatment remains debated due to a potentially increased risk of bleeding.^10^ Several scores predicting the risk of bleeding after reperfusion therapies have been proposed in stroke patients, but it remains unknown whether these scores are applicable in MAS patients. Therefore, the benefits and risks of IVT and EVT treatment of MAS patients must be carefully evaluated. However, offering reperfusion therapy to MAS patients is becoming more common in clinical practice. A large retrospective study including 9,508,804 patients with acute ischemic stroke from 1998 to 2015 in the United States showed that the recanalization therapies were increasingly used among MAS patients. Specifically, IVT utilization was increased from 0.01% in 1998 to 4.91% in 2015, and EVT utilization was increased from 0.05% in 2006 to 1.90% in 2015.^78^ Given the significant progress that was made in EVT since 2015, current numbers of MAS patients that receive EVT could be even higher.^79^, ^80^ According to a retrospective study which compared the effects of recanalization therapies (IVT, EVT, or IVT followed by EVT) in ischemic stroke patients with and without malignancies, no significant differences were found in terms of recanalization rate, 3‐month functional independence, symptomatic intracranial hemorrhage, and mortality rate.^81^ Therefore, active malignancies should not be considered as an absolute contraindication for recanalization therapies. Nevertheless, the reported therapeutic outcomes for IVT or EVT alone are controversial due to potential selection bias, low sample size, and absence of relevant subgroup analyses, for example, the stroke severity and cancer stage.^79^, ^80^, ^82^, ^83^ Further studies on homogenous stroke patient populations who are newly diagnosed with malignancy should be performed, although building such cohorts may take considerable time and effort.

Clinical algorithms for IVT in stroke patients with specific malignancy types have been proposed and highlighted the importance of a personalized approach for each patient.^84^ Malignancy type, existence of prior bleeding history, local tumor invasion, tumor vascularity, and concurrent thrombocytopenia may influence the clinical decision. For instance, IVT in stroke patients concurrent with gastrointestinal (GI) malignancies is generally contraindicated due to increased risk of bleeding in digestive tracts,^85^ worsening the conditions of peptic ulcer, esophageal varices, and erosive lesions. However, adverse outcomes of IVT such as intracranial hemorrhage and mortality rate do not differ between those with and without GI malignancies.^86^ Moreover, bleeding could be controlled with transfusion and fluid resuscitative measures in most cases. Yet additional risk factors should be evaluated before initiating IVT. For stroke patients who are suspected to suffer from hepatic malignancies, portal hypertension, alcoholism, and thrombocytopenia, more risks over benefits should be put on recanalization therapy. Additionally, low hemoglobin count (Hb < 10 g/L) indicates chronic blood loss caused by the tumor itself, which can also increase the risks of recanalization therapy. Current evidence on IVT in hepatic malignancies is far from enough to make a conclusion.

Clinical outcomes of EVT in MAS patients vary depending on different EVT techniques applied. Contact aspiration thrombectomy results in a higher rate of reperfusion (89.3% vs. 64.7%) and first‐pass effect (35.7% vs. 11.8%) as well as shorter procedure time (22 vs. 42 min) compared to stent retriever thrombectomy.^87^, ^88^ However, contact aspiration thrombectomy using smaller‐caliber aspiration devices was less successful in MAS patients, suggesting the thrombi are more difficult to extract.^88^ Analysis of retrieved clots can aid in diagnosis of MAS^89^ since high fibrin/platelet and low erythrocyte contents within a thrombus can suggest MAS.^71^, ^72^

In conclusion, both IVT and EVT are valuable and feasible therapeutic options for MAS patients unless other contraindications exist. However, additional large, randomized trials are required to address relevant questions of intervention safety and efficacy. An individualized treatment approach is recommended based on types of malignancies and stroke severities.^90^, ^91^, ^92^, ^93^

Moreover, MAS patients with Trousseau syndrome typically have poor survival. Therefore, there is a need to determine the effectiveness of rehabilitation treatment and develop a comprehensive treatment strategy earlier than that in the general stroke population. A recent study has found that intensive rehabilitation therapy may be indicated for patients with Trousseau syndrome who are expected to improve physical function after approximately 1 month of rehabilitation.^94^ Besides, retrospective study has found that both antiplatelet and antitumor treatment are recommended to achieve better neurological recovery and oncological prognosis in lung adenocarcinoma patients with Trousseau syndrome.^95^ In other words, antiplatelet and antitumor treatment followed by intensive rehabilitation therapy may be beneficial for MAS patients with Trousseau syndrome.

## 4 THE OPTIMAL CHOICE OF ANTICOAGULATION IN THE PREVENTION OF RECURRENT STROKE

### 4.1 Patients with malignancy in general

Prevention of recurrent ischemic stroke is important in clinical stroke management. The current mainstay of preventive therapy for cardioembolic stroke is anticoagulation.^10^, ^96^ Most cryptogenic strokes are thromboembolic, and thus patients can benefit from anticoagulation therapy.^97^ Oral anticoagulation is effective in preventing atrial fibrillation‐related stroke. It is therefore believed that anticoagulation could reduce stroke recurrence after cryptogenic strokes,^98^ while a considerable socio‐economic impact of suboptimal anticoagulation in high‐risk populations such as malignant patients has been revealed.^99^ In 2018, Navi et al. divided cryptogenic strokes into two subsets which are likely and unlikely to respond to anticoagulation therapy. Corresponding to this idea, malignancy can increase the risk of stroke through several mechanisms, including the hypercoagulability caused by cancer itself, nonbacterial thrombotic endocarditis, iatrogenic effects of chemo/radiotherapy, and tumor embolism.^100^ Indeed, MAS patients have a higher prevalence of deep vein thrombosis or microembolic events than those without malignancy, suggesting venous hypercoagulability.^16^ Anticoagulants may help treat all these conditions, but anticoagulation is not recommended for primary stroke prevention due to the increased bleeding risk.^10^ Currently, it is uncertain as to which form of anticoagulant therapy should be provided to MAS patients.

Heparin, due to its multifaceted biological activity, is a good option for treating malignancy‐associated thrombosis, especially venous thromboembolism (VTE).^101^ Although early administration of unfractionated heparin, low‐molecular‐weight heparin, or heparinoids is not recommended for treatment and prevention of acute ischemic stroke,^102^ subcutaneous heparin prevented stroke recurrence and thrombocytopenia in MAS patients in a small‐scale trial enrolling 19 patients.^103^ However, nine patients discontinued because of medical conditions such as cancer deterioration and unwillingness to continue subcutaneous heparin injection and^103^ long‐term subcutaneous heparin therapy preventing recurrence of MAS based on the results of the trial. A similar situation was reported in the TEACH pilot trial in which 40% of patients who used enoxaparin changed to aspirin later, based on the enrollment failure comparing aspirin and direct oral anticoagulants instead of injectable heparins is recommended to be considered for future clinical trials.^104^ Several studies have been conducted to compare the efficacy and safety of other anticoagulants in preventing recurrence of MAS, including nonvitamin K anticoagulants (NOACs), warfarin, or aspirin (Table 1). The rates of ischemic stroke recurrence and major bleeding events are similar in cancer patients who receive NOACs, warfarin, heparin, or aspirin. However, the reliability of these results was debatable due to the small sample size in these trials^104^, ^105^, ^106^, ^107^ except for the NAVIGATE ESUS trial which involved 543 patients,^107^ in which reduced stroke was not observed by the administration of 15 mg rivaroxaban per day compared with that of aspirin in patients with embolic stroke of an undetermined source. Additional prospective studies enrolling larger number of patients are highly warranted.

**TABLE 1:** Summary of existing studies reporting safety and efficacy of anticoagulant use in malignancy‐associated stroke patients.

Study (First Author‐year)	Study type	Anticoagulants	No. of patients	Efficacy outcome results (*p* value)	Safety outcome results (*p* value)
Jang‐2015^98^	Retrospective single‐center observational study	Enoxaparin vs. warfarin	79 (29 vs. 50)	0.249	0.960
Nam‐2017^99^	Retrospective bicentric observational study	NOAC vs. LMWH	48 (7 vs. 41)	0.846	0.696
Navi‐2018^97^	Randomized clinical trial	Enoxaparin vs. aspirin	20 (10 vs. 10)	>0.05	>0.05
Majander‐2019^100^	Randomized clinical trial	Rivaroxaban vs. aspirin	543 (254 vs. 289)	0.3137	0.9539

*Note*: “Efficacy” outcome refers to ischemic stroke; “Safety” outcome refers to major bleeding events.

Abbreviations: IS, ischemic stroke; LMWH, low molecular weight heparin; NOAC, nonvitamin K oral anticoagulant.

D‐dimers, next to predicting MAS, can be utilized for anticoagulant monitoring, assessing the efficacy of anticoagulant therapy. Patients treated with enoxaparin showed a significant decrease in D‐dimer level compared to warfarin‐treated patients (17.06 to 3.88µg/mL vs. 17.78 to 17.42µg/mL; *p* = 0.026).^105^ Consequently, heparin may perform better than warfarin in the secondary prevention of MAS. The Khorana score (Table S2) might be a feasible method to predict the risk of recurrent ischemic stroke in MAS patients, given its proven validity in evaluating VTE risk.^108^ However, studies specifically designed to investigate its efficacy in MAS patients are required.

### 4.2 Malignancy concurrent with AF

Application of anticoagulant strategies in malignant patients with AF is an important topic, but no guidelines are available in this regard. Furthermore, most MAS patients are cryptogenic but not cardioembolic,^109^ so the right strategies for stroke prevention in malignant patients are a matter of ongoing debate.

The CHADS_2_ (Table S3) and CHA_2_DS_2_‐VASc (Table S4) scores are risk stratification schemes to help determine whether clinicians should initiate anticoagulant therapy, taking into account age, sex, history of congestive heart failure, hypertension, diabetes and stroke, and vascular diseases. However, active malignancy is not included in the scoring systems, and studies showed that these scoring systems are not expected to predict ischemic stroke risk in patients with malignancies and recently diagnosed AF.^31^, ^110^ As a result, Sorigue and Miljkov proposed an algorithm combining CHA_2_DS_2_‐VASc scores and bleeding risk factors to decide whether patients with ESUS malignancies should receive anticoagulation or whether the risk of bleeding is too high. Bleeding risk factors include major factors as gastrointestinal mass, previous major bleeding, concomitant antiplatelet treatment, thrombocytopenia (<50,000/μL), and time in therapeutic range, and minor factors such as age > 80 years, metastatic disease, thrombocytopenia (<100,000/μL), renal failure (glomerular filtration rate < 30 mL/min/1.73m^2^), and drug interactions. Anticoagulation is recommended for those with a moderate bleeding risk and high thromboembolic risk (CHA_2_DS_2_‐VASc > 6).^34^ In light of the causative association between recent onset AF and malignancy, eliminating external triggers such as surgery, sepsis, or hypoxemia may be the best way to address secondary AF. Overall, new triggers should be addressed first, followed by reducing the risk of thrombosis and bleeding.

Warfarin and nonvitamin K anticoagulants (NOACs, apixaban, rivaroxaban, edoxaban, and dabigatran) have been thoroughly studied regarding safety and efficacy in malignant patients with AF.^111^, ^112^, ^113^, ^114^, ^115^, ^116^, ^117^ Although results varied (Table 2), it can be concluded that NOACs appear at least as safe and effective as warfarin in preventing ischemic stroke in MAS patients with AF, with an thromboembolic rate of 0%–4.9% and bleeding rate of 1.2%–4.4% per year.^111^ In conclusion, an individualized and adaptable approach tailored to the individual patient is needed until high‐quality evidence becomes available.

**TABLE 2:** Study characteristics comparing nonvitamin K anticoagulants and warfarin for anticoagulation therapy in malignant patients with atrial fibrillation.

Study (First Author‐Year)	Study Type	NOACs	No. of NOACs/Warfarin users	Malignancy types	Follow up (month)	Efficacy results (ischemic stroke)	Safety results (major bleeding)
Fanola‐2018^105^	Post hoc analysis from ENGAGE AF‐TIMI 48 trial	Edoxaban	750/395	Prostate (13.7%), breast (6.5%), bladder (7.5%), gastrointestinal (20.5%), lung or pleura (11%), skin (5.9%), pancreatic (3.8%), liver, gallbladder, or bile ducts (3.8%), esophageal (2.5%), oropharyngeal (2.6%), renal (2.5%), uterine (2.1%), brain (2.1%), genital (1.3%), thyroid (1.1%), leukemia (2.8%), lymphoma (2.2%), others (1.3%), unspecified cancer type (1.5%)	33.6	Annualized rate (1.22% vs. 2.08%, *p* = 0.12)	Annualized rate (7.82% vs. 8.18%, *p* = 0.31)
Kim‐2018^106^	Retrospective population‐based cohort study	Dabigatran, rivaroxaban, apixaban	388/388	Stomach (20.6%), colorectal 7 (14.9%), thyroid (10.8%), prostate (9.3%), lung (12.2%), melanoma (5.9%), biliary tract (5.4%), urinary tract (6.1%), genitourinary (12.2%), head and neck (4.1%), hepatocellular carcinoma (3.0%), breast (2.4%), ovary and endometrial (2.6%), renal cell carcinoma (3.1%), hematologic malignancy (2.2%), others (3.2%)	21.6	Annualized IS/SE rate(1.3% vs. 5.9%, *p* < 0.001)	Annualized major bleeding rate (1.2% vs. 5.1%, *p* < 0.001)
Shah‐2018^107^	Retrospective population‐based cohort study	Dabigatran, rivaroxaban, apixaban	6084/10021	Breast (19.2%), gastrointestinal (12.7%), lung (12.3%), genitourinary (29.2%), gynecological (2.4%), hematological (9.8%), others (14.4%)	12	Dabigatran: HR 0.89, *p* = 0.63; Rivaroxaban: HR 0.74, *p* = 0.35; Apixaban: HR 0.71, *p* = 0.6	Dabigatran: HR 0.96, *p* = 0.75; Rivaroxaban: HR 1.09, *p* = 0.59; Apixaban: HR 0.37, *p* = 0.01
Chen‐2019^108^	Post hoc analysis from ROCKET AF trial	Rivaroxaban	309/331	Prostate (28.6%), breast (14.7%), colorectal (16.1%), gastrointestinal (3%), lung (3.1%), melanoma (5.9%), leukemia or lymphoma (5.2%), gynecological (6.6%), genitourinary (12.2%), head and neck (3.9%), thyroid (2.5%), brain (0.3%), others (3%), unspecified cancer type (3.9%)	22.8	HR 0.52, *p* = 0.21	HR 1.09, *p* = 0.79
Konrad‐2019^109^	Retrospective single‐center cohort study	Dabigatran, rivaroxaban, apixaban	44/−	Colorectal	28.5	5 of 44 (11.4%)	2 of 44 (4.5%)
Atterman‐2019^110^	Retrospective single‐center cohort study	Not reported	506/1012	Gastrointestinal (23.8%), pancreatic (2.0%), lung (8.5%), breast (8.3%), gynecological (5.1%), urological (31.7%), prostate (22.4%), intracranial (1.5%), hematological (10.3%), others (13.0%)	28.8	HR 0.66, *p* = 0.000	HR 1.01, *p* = 0.840

*Note*: “Efficacy” outcome refers to ischemic stroke; “Safety” outcome refers to major bleeding events.

Abbreviations: LMWH, low molecular weight heparin; NOAC, nonvitamin K oral anticoagulant.

## 5 CONCLUSION

Malignancy increases the risk of ischemic stroke, and MAS patients have worsened prognosis than stroke patients without malignancy. Therefore, early identification and management of MAS patients is critical to mitigate the burden generated from both diseases. In turn, occult malignancies should be suspected in every patient presenting with cryptogenic stroke especially when there is absence of typical stroke risk factors, increased level of inflammation, hypercoagulability, and lesions in multiple vascular territories. Combination of established scoring systems can increase the sensitivity and specificity of the diagnostic workup. Active malignancies should not be considered an absolute contraindication for recanalization therapies. Atrial fibrillation is often concurrent with malignancies, increasing the risk of ischemic stroke. The optimal anticoagulant strategy for MAS patients, especially those with atrial fibrillation, remains uncertain, and high‐quality evidence from clinical trials is highly warranted.

## FUNDING INFORMATION

P.L. is supported by the National Natural Science Foundation of China (NSFC, U22A20295, 91957111, 81971096, 82061130224, and M‐0671), New Frontier Technology Joint Research (SHDC12019102) and Ward Building Project for Demonstration and Research sponsored by Shanghai Shenkang Hospital Development Center, Shanghai Municipal Education Commission‐Gaofeng Clinical Medical Grant Support (20181805), “Shuguang Program” supported by Shanghai Education Development Foundation and Shanghai Municipal Education Commission (20SG17), “Shanghai Outstanding Academic Leaders Program” from Shanghai Municipal Science and Technology Committee (20XD1422400), and the Institutional Clinical Research Program (PYII20‐03). P.L is also supported by the Innovative Research Team of High‐level Local Universities in Shanghai (SHSMU‐ZLCX20211602). P.L. and J.B. are supported by a Newton Advanced Fellowship grant provided by the UK Academy of Medical Sciences (NAF\R11\1010).

## CONFLICT OF INTEREST STATEMENT

The authors declare no potential conflicts of interest with respect to the authorship, and/or publication of this article. Johannes Boltze and Peiying Li are Editorial Board members of CNS Neuroscience and Therapeutics and co‐authors of this article. To minimize bias, they were excluded from all editorial decision‐making related to the acceptance of this article for publication.

## Supporting information


Appendix S1

## Data Availability

Data sharing not applicable to this article as no datasets were generated or analysed during the current study.
