# Supplementary material for: Malignancy‐associated ischemic stroke: Implications for diagnostic and therapeutic workup
Source: CNS Neurosci Ther. 2024 Mar 26;30(3):e14619. doi: 10.1111/cns.14619 (PMC10965754; doi:10.1111/cns.14619)
Supplement: Supplementary file 1 — Appendix S1 [file CNS-30-e14619-s001.docx]

**Supplementary Table 1 Trousseau scoring system for malignancy-associated stroke (MAS)^1^**

| Item | Odds ratio | Points |
| --- | --- | --- |
| D-dimer≥10.0μg/ml | 34.04 | 2 |
| Lesions in multiple territories* | 13.89 | 2 |
| Active cancer^†^ | 4.02 | 1 |
| PLT<150,000/μL | 3.53 | 1 |
| Sex: female | 2.24 | 1 |
| Trousseau score (total sum score) ≥3^‡^ | MAS |  |

*Multiple territories: both hemispheric, or both supra- and infra-tentorial regions

^†^Active cancer: histopathologically confirmed malignancy within the previous 6 months, ongoing cancer treatment, local recurrence, or metastasis

^‡^Score 3 was decided by receiver operating characteristic curve and logistic regression analysis for predicting cryptogenic stroke

**Youden’s index**

The formula for the index^2^ of performance is:

$$J=\frac{ad-bc}{(a+b)(c+d)}$$

where, of (a+b) diseased patients, “a” is correctly diagnosed and “b” is false negatives, and where of (c+d) controls, “d” is correctly reported and “c” is false positives.

**Supplementary Table 2 Khorana scoring system for venous thromboembolism (VTE)^3^**

| Patient Characteristic | Risk Score |
| --- | --- |
| Site of cancer |  |
| Very high risk (stomach, pancreas) | 2 |
| High risk (lung, lymphoma, gynecologic, bladder, testicular) | 1 |
| Prechemotherapy platelet count≥350,000/mm^3^ | 1 |
| Hemoglobin<10g/dL or use of red cell growth factors | 1 |
| Pre-chemotherapy leukocyte>11000/mm^3^ | 1 |
| Body mass index≥35kg/m^2^ | 1 |

Score=0: in the low-risk category; Score=1 to 2: in the intermediate-risk category; Score≥3: in the high-risk category

**Supplementary Table 3 CHADS_2_ index system for stroke^4^**

| Item | Points |
| --- | --- |
| **C**ongestive heart failure | 1 |
| **H**ypertension | 1 |
| **A**ge 75 years or older | 1 |
| **D**iabetes mellitus | 1 |
| **H**istory of stroke or transient ischemic attack | 2 |

Score=0 to 1: in the low-risk category; Score=2 to 3: in the intermediate-risk category; Score≥4: in the high-risk category

**Supplementary Table 4 CHADS_2_ -VASc index system for stroke in patients with atrial fibrillation^5^**

| Item | Points |
| --- | --- |
| **C**ongestive heart failure or left ventricular dysfunction | 1 |
| **H**ypertension | 1 |
| **A**ge≥75 years | 2 |
| **D**iabetes mellitus | 1 |
| **H**istory of stroke or transient ischemic attack or thromboembolism | 2 |
| **V**ascular disease (prior myocardial infarction, peripheral artery. disease, or aortic plaque) | 1 |
| **A**ge 65-74 years | 1 |
| **S**ex **c**ategory (ie female gendar) | 1 |

Score=0: in the low-risk category; Score=1: in the intermediate-risk category; Score≥2: in the high-risk category

**References**

1. Hasegawa Y, Setoguchi T, Sakaida T, Iuchi T. Utility of a scoring system for differentiating cancer-associated stroke from cryptogenic stroke in patients with cancer. *Neurol Sci*. May 2020;41(5):1245-1250. doi:10.1007/s10072-019-04231-5

2. Youden WJ. Index for rating diagnostic tests. *Cancer*. Jan 1950;3(1):32-5. doi:10.1002/1097-0142(1950)3:1<32::aid-cncr2820030106>3.0.co;2-3

3. Khorana AA, Kuderer NM, McCrae K, et al. Cancer associated thrombosis and mortality in patients with cancer stratified by khorana score risk levels. *Cancer Med*. Nov 2020;9(21):8062-8073. doi:10.1002/cam4.3437

4. Gage BF, Waterman AD, Shannon W, Boechler M, Rich MW, Radford MJ. Validation of clinical classification schemes for predicting stroke: results from the National Registry of Atrial Fibrillation. *JAMA*. Jun 13 2001;285(22):2864-70. doi:10.1001/jama.285.22.2864

5. Lip GY, Nieuwlaat R, Pisters R, Lane DA, Crijns HJ. Refining clinical risk stratification for predicting stroke and thromboembolism in atrial fibrillation using a novel risk factor-based approach: the euro heart survey on atrial fibrillation. *Chest*. Feb 2010;137(2):263-72. doi:10.1378/chest.09-1584
